# Supplementary material for: Detailed molecular and epigenetic characterization of the pig IPEC-J2 and chicken SL-29 cell lines
Source: iScience. 2023 Feb 20;26(3):106252. doi: 10.1016/j.isci.2023.106252 (PMC10018572; doi:10.1016/j.isci.2023.106252)
Supplement: Data S2. Complete homer output for identified motifs in Chicken SL-29, related to Tables 5 and 6 — Homer motif analysis results for histone modifications H3K4me1, H3K4me3, H3K27ac, enhancers, and ATAC-seq of chicken SL-29 cell line. Parameters for possible false positives is as mentioned earlier for S5. [file mmc3.zip › Data_S2/S6/Chicken_SL_29/peak_files_CTCF/homerResults/motif2.similar.html]

motif2

## Information for motif2

A
T
G
C
A
G
T
C
T
C
G
A
A
G
T
C
C
G
A
T
A
C
G
T
A
T
G
C
A
T
G
C
A
C
T
G
T
A
C
G
  
Reverse Opposite:  

A
T
G
C
T
G
A
C
T
A
C
G
T
A
C
G
T
G
C
A
G
C
T
A
T
C
A
G
A
G
C
T
T
C
A
G
T
A
C
G
  

|  |  |
| --- | --- |
| p-value: | 1e-356 |
| log p-value: | -8.207e+02 |
| Information Content per bp: | 1.474 |
| Number of Target Sequences with motif | 9022.0 |
| Percentage of Target Sequences with motif | 30.60% |
| Number of Background Sequences with motif | 5993.9 |
| Percentage of Background Sequences with motif | 20.60% |
| Average Position of motif in Targets | 149.0 +/- 81.2bp |
| Average Position of motif in Background | 150.1 +/- 93.7bp |
| Strand Bias (log2 ratio + to - strand density) | 0.0 |
| Multiplicity (# of sites on avg that occur together) | 1.35 |
| Motif File: | file (matrix) reverse opposite |

### Similar de novo motifs found

|  |  |  |  |  |  |  |  |
| --- | --- | --- | --- | --- | --- | --- | --- |
| Rank | Match Score | Redundant Motif | P-value | log P-value | % of Targets | % of Background | Motif file |
| 1 | 0.907 | C T G A A G T C G C A T C G A T A G T C G A T C A C T G A T C G | 1e-345 | -796.486496 | 33.95% | 23.68% | motif file (matrix) |
| 2 | 0.925 | A T G C C T A G A G C T C G A T G A C T G A T C A T G C C A T G | 1e-274 | -631.267479 | 11.07% | 5.71% | motif file (matrix) |
| 3 | 0.769 | C G T A C A T G A C G T G T A C C G T A A G C T A C G T A C G T A G T C A G T C A C G T A C T G G A C T | 1e-180 | -414.818584 | 8.16% | 4.36% | motif file (matrix) |
| 4 | 0.791 | A T G C A T C G A G C T A T G C C T G A A G T C C G A T C G A T G T A C A G T C A T C G A T C G A G T C | 1e-161 | -371.451163 | 4.67% | 2.06% | motif file (matrix) |
| 5 | 0.670 | G T A C T C A G T A C G A C T G C T G A T C G A C G T A G C A T | 1e-137 | -317.209572 | 28.09% | 21.88% | motif file (matrix) |
| 6 | 0.715 | G C A T G A C T A T G C A T G C C A G T T G A C A G T C A T G C | 1e-101 | -234.813322 | 35.08% | 29.29% | motif file (matrix) |
| 7 | 0.630 | A C G T A C G T T G A C A G T C A T G C A C T G A G T C A G C T | 1e-97 | -223.836549 | 9.22% | 6.09% | motif file (matrix) |
| 8 | 0.773 | A T G C A T G C A T C G A C G T C G A T G T A C A G T C A T C G A T C G A G T C T A C G A T G C A T G C | 1e-89 | -204.934110 | 23.50% | 18.80% | motif file (matrix) |
| 9 | 0.636 | T A C G T G C A A C T G T C A G A T C G T A G C T C A G A C T G T C G A T G C A | 1e-65 | -151.269811 | 31.31% | 26.81% | motif file (matrix) |
| 10 | 0.612 | G T C A A G T C A G C T A C G T G A T C A G T C A G T C A C T G G A T C G T A C | 1e-56 | -130.008601 | 1.84% | 0.85% | motif file (matrix) |
| 11 | 0.695 | A T G C A C G T A G C T A G T C A G T C T C A G A G C T A G C T A G C T T A G C A G T C A C T G A T C G | 1e-52 | -121.991606 | 0.40% | 0.06% | motif file (matrix) |
